# A facility location model for analysis of current and future demand for sexual health services

**DOI:** 10.1371/journal.pone.0183942

**Published:** 2017-08-29

**Authors:** Rudabeh Meskarian, Marion L. Penn, Sarah Williams, Thomas Monks

**Affiliations:** 1 NIHR CLAHRC Wessex Methodological Hub, Faculty of Health Sciences, University of Southampton, Southampton, United Kingdom; 2 Solent NHS Trust Research, Southampton, United Kingdom; University of Texas at Dallas, UNITED STATES

## Abstract

In this paper we address the clinic location selection problem for a fully integrated Sexual Health Service across Hampshire. The service provides outpatient services for Genito-Urinary Medicine, contraceptive and reproductive health, sexual health promotion and a sexual assault referral centre. We aim to assist the planning of sexual health service provision in Hampshire by conducting a location analysis using both current and predicted patient need. We identify the number of clinic locations required and their optimal geographic location that minimise patient travel time. To maximise the chances of uptake of results we validate the developed simple algorithm with an exact method as well as three well-known, but complex meta-heuristics. The analysis was conducted using car travel and public transport times. Two scenarios were considered: current clinic locations only; and anywhere within Hampshire. The results show that the clinic locations could be reduced from 28 to 20 and still keep 90% of all patient journeys by public transport (e.g. by bus or train) to a clinic within 30 minutes. The number of clinics could be further reduced to 8 if the travel time is based on car travel times within 15 minutes. Results from our simple solution method compared favourably to the exact solution as well as the complex meta-heuristics.

## Introduction

In the UK, sexual health covers the provision of advice and services around contraception, relationships, sexually transmitted infections (STIs) (including HIV) and termination of pregnancy. The importance of access to such services is demonstrated by the various policy changes in England sought to improve access to sexual healthcare [1]. However, there is still room for improvement, in England during 2011, one person was diagnosed with HIV every 90 minutes, almost half the adults diagnosed were passed the point at which they should have started treatment [2]. In 2010, England was in the bottom third of 43 countries in the World Health Organization’s European Region and North America for condom use among sexually active young people.

Evidence demonstrates that sexual health interventions and service outcomes could be improved through effective commissioning. For instance, For every £1 spent on contraception, £11 is saved in other healthcare costs [3]. Early testing and diagnosis of HIV reduces treatment costs—£12,600 per annum per patient, compared with £23,442 with a later diagnosis [4]. The pressure on capacity of such services continues to grow due to high demand owing to an increase in the prevalence of many STIs (Sexually transmitted illnesses). This paper discusses the issues surrounding the location of sexual health service facilities and the needs of those who must plan for these services. To meet this need, a flexible practical location analysis model is developed, a description of which is presented here. We will show that it is possible to build a generic model capturing the underlying process of patients traveling to clinics. The results are represented using a Geographic Information System (GIS), illustrating on a map the clinics and information on the population they serve. The model provides the necessary information for answering a variety of “what if?” questions such as the effect of changing the location of the clinics, opening or closing clinics.

It is evident that healthcare infrastructure is essential for effective operations of healthcare systems. An efficient facility location can save cost and improve the facility utilization. High costs associated with property acquisition and facility construction make facility location or relocation projects long-term investments. Decision makers must select sites that will not simply perform well according to the current system state, but that will continue to be effective as populations shift.

This project aims to:

1. Forecast future demand for different aspects of the service over the next 3–5 years, specifically for the period 2018–2020 as well as supporting immediate service configuration.
2. Perform a facility location analysis based on the current and forecasted demand to identify an optimal number of clinics and their geographic location. The project aims to reduce the number of clinics while maintaining/improving service access, as such the problem will be formulated as a maximal coverage problem and solved by a greedy algorithm.
3. Validating the developed greedy algorithm through comparison with four alternative methods of solving real life facility location problems and analyse the advantages/limitations of more complicated methods compared to the simple greedy algorithm in such cases.
4. The aim is not to build a single image of the future provision of services but rather to allow participating organizations to explore a number of possible futures through use of a decision making tool.

## Location analysis

The role of quantitative location analysis in planning services in healthcare is well documented [5–7]. It provides a framework for investigating service accessibility problems, comparing the quality (in terms of efficiency) of previous locational decisions, and generating alternatives either to suggest more efficient service systems or to improve existing systems.

A location-allocation model is a method for finding optimal sites for facility locations. The method involves simultaneously selecting a set of locations for facilities and assigning spatially distributed sets of demands to these facilities to optimise some specified measurable criterion. Predictions on patient numbers, travel times and travel distances are important to answer questions on the number of health centres to provide, the services they offer, and the geographical location of these centres.

In what follows, we focus on discrete facility location models, as opposed to the class of continuous location models since they have been used far more extensively in health care location problems [8]. Discrete location models assume that demands can be aggregated to a finite number of discrete points. Thus, we might represent a city by several hundred or even several thousand points or nodes (e.g. postcodes). Similarly, discrete location models assume that there is a finite set of candidate locations or nodes at which facilities can be sited (e.g. current locations or possible postcodes).

Many location models have been formulated that can be applied to the location of healthcare facilities. The p-median model in [9, 10] identifies optimal locations of p facilities by minimising total weighted travel distance from each demand node to the nearest facility. With increasing average travelling distance, facility accessibility decreases, and thus the location's effectiveness decreases. A major drawback of these models is that distances may be unreasonably long for people living in less accessible places within a target area. Solutions that aim to minimize total travel distance alone, such as p-median, may be inequitable forcing some users to travel far [7]. The P-centre model addresses the problem of needing too many facilities to cover all demand by relaxing the service standard. This model finds the location of p facilities to minimise the coverage distance while covering all demand. Daskin [11] provided a traditional formulation of this problem while Elloumi, Labbe and Pochet [12] presented an alternative formulation.

The problems described above can be used to locate a wide range of public and private facilities. However, in some cases such as healthcare where coverage is the main concern minimising the average distance travelled may not be an appropriate approach. A demand is set to be covered if it can be served within a specified time. The literature on covering problems is divided into two major parts, location set covering problem where coverage is required and the maximal covering problem where coverage is optimised. For a detailed review see [13, 14]. In the set covering problem the objective is to minimise the cost of facility location such that a specified level of coverage is obtained. Effectively the set covering problem examines the number of clinics needed to guarantee a certain level of coverage to all demand nodes. It makes no distinction between demand nodes based on demand size which can lead to more centres being required than is feasible as such they are mostly suitable for emergency services [5]. As an alternative, the location goal can be shifted so that the facility location are selected in a way to give as many patients/customers as possible the desired level of coverage. This new goal is that of the maximal covering problem.

The maximal covering problem (MCP) introduced by Church and ReVelle [15] maximises the population covered for a fixed number of facilities. There have also been further MCP models with a constraint that ensures that no demand is further away from a facility than a set service distance. One of the key assumptions of the MCP is that coverage is binary, that is, a certain patient location is either fully covered (if there is a facility within distance S from patient’s location), or not covered at all. For more information on various location selection models see [11, 14, 16] and references therein.

In this paper we concentrate on a variation of the MCP model to select maximum *P* facilities that would provide coverage within traveling time *T* from patient’s location.

## Methods

This project was carried on as a service evaluation and only used anonymised data recorded routinely by the service provider. As such this project, did not require individual patient consent. This was approved by the ethics committee. No patients were involved or identified, no new data were generated or collected, and no care pathways were altered. This project is registered as service evaluation at NHS Solent Trust and has been approved by the University of Southampton Ethics and Research Governance Committee (submission ID 23704).

### Study setting

**Fig 1:**
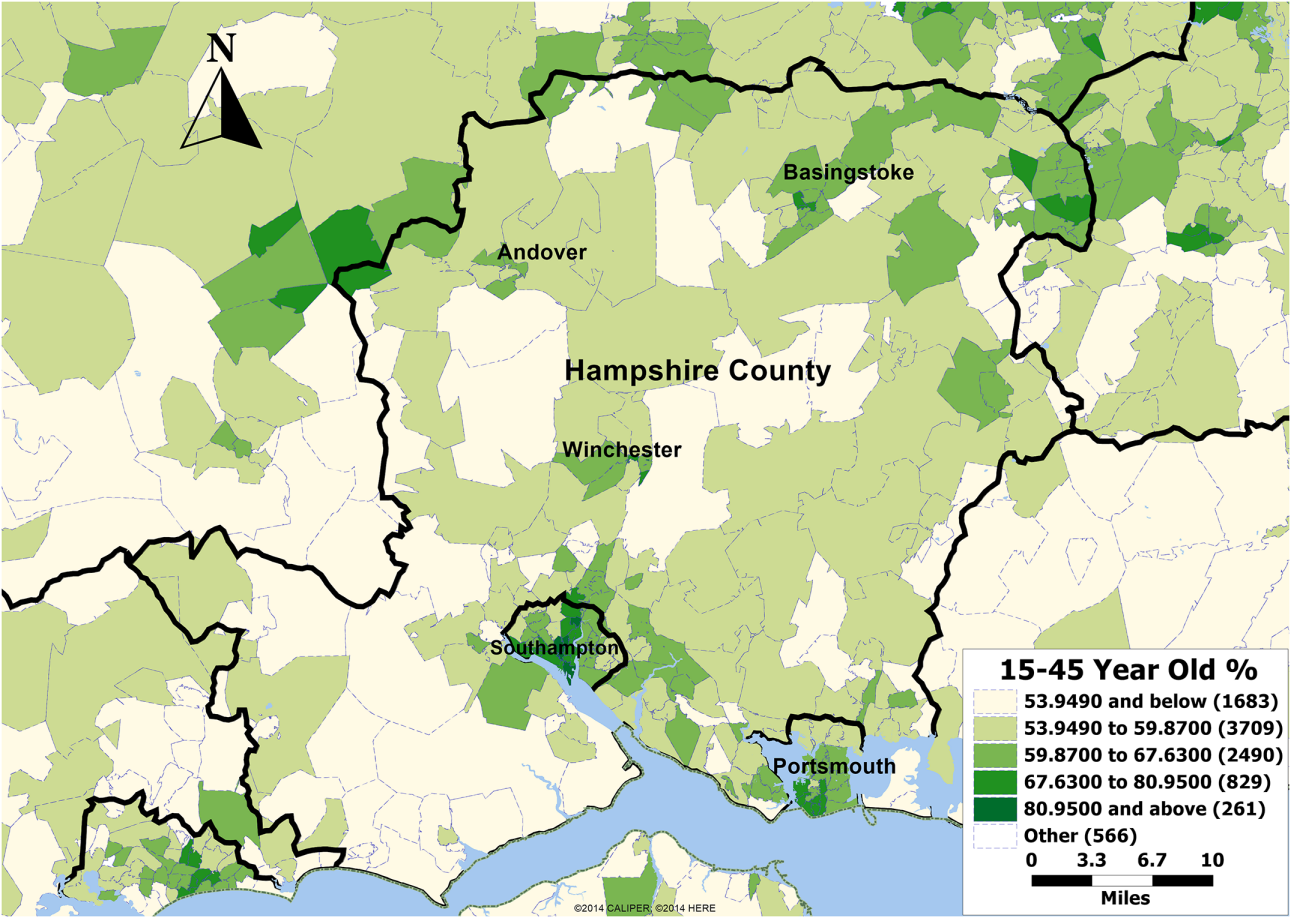
Hampshire map showing the population of 15–44 year old.

Hampshire is a county on the southern coast of England in the United Kingdom. It is the most populous county in the United Kingdom (excluding the metropolitan counties) with almost half of the county's population living within the South Hampshire conurbation which includes the cities of Southampton and Portsmouth. The total population as in 2011 census is 1,759,800 of which 696,799 are between 15 and 44 (the age group most likely to use sexual health services) Fig 1.

There are two main types of clinic providing sexual health services across Hampshire, central hubs and smaller spokes. The central hubs are located in high population areas and are open every weekday, while the spokes are spread out across smaller population areas and have a variety of opening times. Furthermore, there are clinics held at schools and colleges that cover both a health promotion remit as well as appointments however these are only intended for the students within these establishments and have historically low demand.

In health care, the implications of poor location decisions extend well beyond cost and customer service considerations. If too few facilities are utilized and/or if they are not located well, increases in morbidity (infections) can have a major impact on public health. Thus, facility location modelling takes on an even greater importance when applied to the siting of health care facilities.

Since the project partners were interested in a reduction in the number of clinics commissioned while maintaining/improving access for patients, we concentrate on the maximal covering problem [15]. This model takes into account the fact that if we cannot cover all demands because the cost of doing so is prohibitive, we would prefer to cover those demand nodes that generate a lot of demand rather than those that generate relatively little demand.

### Problem formulation

Let *T* be the maximum acceptable travel time to a clinic. We define the indicator variable as follows:

$$a_{ij}=\{\begin{array}{ll} 1 & \text{if demand }i\text{ is covered by a facility at site }j\text{ within }T\text{ travel time},\\ 0 & \text{Otherwise}. \end{array}$$

Further, we define the following sets and inputs:

*I* = set of demand nodes

*J* = set of candidate facility sites

*h*_*i*_ = *demand at node i*

*P* = number of facilities to locate

In addition, we require the following decision variables

$$X_{j}=\{\begin{array}{ll} 1 & \text{if a facility is located at site }j,\\ 0 & \text{if not}. \end{array}$$

$$Z_{i}=\{\begin{array}{ll} 1 & \text{if demand node }i\text{ is covered},\\ 0 & \text{if not}. \end{array}$$

The maximal covering location problem can be formulated as follows:

$$\text{Maximise}\;\sum_{i\in I}h_{i}Z_{i}$$ (1)

$${\text{Subject to}\;\text{Z}}_{\text{i}}-\sum_{j\in J}a_{ij}X_{j}\leq 0,\;\forall i\in I,$$ (2)

$$\sum_{j\in J}X_{j}\leq P,$$ (3)

$$X_{j}\in \left\{0,1\right\},\;\forall j\in J,$$ (4)

$$Z_{i}\in \left\{0,1\right\},\;\forall i\in I.$$ (5)

The objective function (1) maximizes the number of covered demands. The constraint (2) states that demand node *i* cannot be counted as covered unless we locate at least one facility that is able to cover the demand node. The constraint (3) states that at most *P* facilities are to be located (P is increased incrementally until the desired (maximum) coverage level is achieved) and the last two constraints (4) and (5) are standard integer constraints i.e. these variables can only take the values 0 or 1.

The UK postcode is usually in the form of (SO17 1BJ: SO (Area) 17 (District) 1(Sector)). In our analysis, the demand is aggregated at postcode sector level (e.g. SO17 1). This is inevitable as any further aggregation such as postcode area would be too general and cover a wide geographical area while on the other hand, due to anonymity requirements full postcodes are not available. As for the potential location of facilities, where the exact location is known we have used a full postcode e.g. current clinics locations, otherwise centroids of the postcode sectors in Hampshire are considered as potential locations.

### Solution procedures

Typically, these problems once formulated are large-scale optimization models that are difficult to solve in a reasonable amount of time. Two types of approaches are used to solve the facility location models: the *exact solution approach* [17] and *heuristic approach* [18]. Various heuristics have been developed in the literature for solving this type of problem. Murray et al [19] applied simulated annealing to location selection models. Reveller et al [20] applied meta-heuristics to large maximal covering location problem with high percentage coverage, while Tong et al [21] solved a regional service coverage maximisation problem through a developed genetic algorithm.

In order to solve a model with a large number of constraints and variables, a heuristic approach is developed here. This can produce acceptable solutions with fewer computational resources but will not guarantee finding the best solution.

We have investigated five different solution methods for solving the MCP: 1) a greedy algorithm, 2) a genetic algorithm, 3) a tabu search method and 4) a simulated annealing algorithm 5) an exact solution method based on Gurobi [22]. The validity of these methods are tested against the set problems considered in [23], which can be accessed through the link provided in [24].

We aim to study the limitations of the greedy algorithm through a comparison with the methods given above in solving real life facility location problems. Including, identifying the comparative advantages and limitations of more complicated methods over simple greedy algorithm in such cases.

Below we present a description of the implemented greedy algorithm, we refer the reader to Supplementary Information for a detailed description of the three alternative heuristic approaches.

A greedy algorithm is a mathematical process that looks for simple, easy-to-implement solutions to complex, multi-step problems by deciding which next step will provide the most benefit. It is important to note that the greedy algorithm never removes facility sites from the solution set. Therefore, it is possible that a facility site added to the solution set in the early iterations of the algorithm may not be justified later in the algorithm due to subsequent facility site assignments. The presence of a "no longer justified" site in the solution set would imply non-optimality. In our greedy routine, total demand covered by each candidate location is calculated. The candidate facility that covers the most demand within a given travel time *T* is selected as a suggested facility. The demand covered by this chosen facility is deducted from the overall demand and the processes is repeated until no unmet demand remains.

## Data

### Data requirements

When exploring future scenarios of patient care, and corresponding location of centres, health planners will need to consider the following options:

#### Mode of transport

Patients use different modes of transport to complete the journey to a clinic. A patient will usually travel by bus, train, car, bicycle, or on foot. Travel considerations are typically influenced by location of the patient. A patient living in a rural area, with limited public transport might, for example, travel by car, whereas a city dwelling patient is more likely to make use of the public transport network.

#### Demand for services

Patient demand is estimated for the different services across the defined population area. For example, the number of patients expected to visit a sexual health clinic. Forecasts of demand will need to account for demographic and migration change.

When planning services in healthcare for long term sustainability, forecasts of demand play a crucial role. It has been shown that projections become increasingly uncertain the further they are carried forward [25]. In general, fertility levels tend to be over-projected and net migration and improvements in life expectancy are under-projected introducing an uncertainty into the projected values.

### Data sources

In order to provide accurate location analysis and a useful scenario analysis tool the following information is required:

- Travelling times for both public transport and driving for all patients travelling to a given clinic; the driving times were calculated using GIS software (Maptitude) [26] with a built-in add on (MileCharter) [27]. While public transport times were extracted from the Google API application [28].
- Historical and future demand; Here, the future demand is forecasted based on the Hampshire population projection data published by Hampshire County Council [29]. Historical demand in 2015 is taken as the baseline and then the demand for 2018 and 2020 are forecast considering the projected changes to the population by age and gender. The change in population over the 3–5 years considered is very small as the periods considered are relatively short.

Routinely collected data on service provision was provided for the period March 2014 to November 2015 which contained 203,856 appointments, 68 clinic locations (some were temporary or not currently operational). Out of these only 28 have significant demand and are held regularly. For this reason and as instructed by the planners, in what follows, for comparison purpose we have only concentrated on the analysis of these 28 clinic locations. The data included details on appointment location and date, individual patient’s information such as partial address, age and gender as well as diagnosis and service data.

## Scenarios

The following scenarios were considered after long discussions with the public health planners in Hampshire. The maximum travel times of 30 minutes for public transport and 15 minutes for driving time were specifically selected based on the historical weighted average travel time of patients and was further confirmed by the planners:

1. **Existing Clinic Locations Only—Projected Demand**
  Under this scenario the current clinic locations are the only candidate facilities to consider.
  - Within 30 minutes of public transport links to a clinic.
  - Within 15 minutes driving time of a clinic.
2. **Postcode Sectors and Clinic Locations—Projected Demand**
  Under this scenario all postcode sectors within Hampshire as well as the current clinic locations are considered.
  - Within 30 minutes of public transport links to a clinic.
  - Within 15 minutes driving time of a clinic.

## Results

The solution methods except for the exact method which is implemented in Python 3.5.2 were coded in MATLAB 2016a installed on a Dell laptop running Windows 8.1, with an i7 processor and 16 GB of memory. These tests we run during the period May-July 2016.

**Fig 2:**
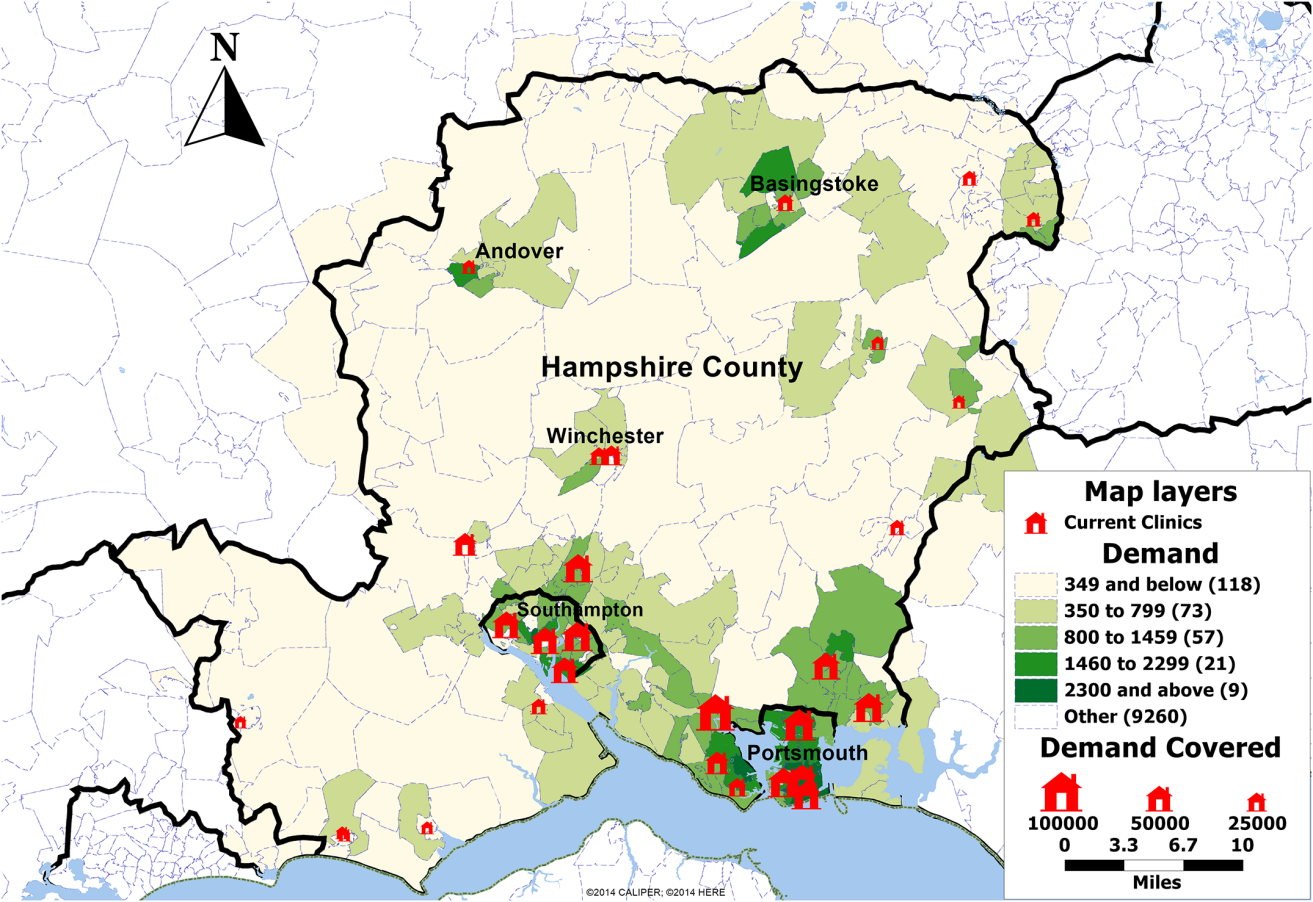
Map showing the demand by postcode sector in Hampshire, UK and current clinics (red). The size of the clinics is proportional to the demand expected over the period of one year.

Under scenario 1 there are 278 demand points (postcode sectors) each with a particular projected demand. There are 28 candidate locations (shown on map in Fig 2) which include all the hubs and medium size clinics that are currently operating. The problem was solved by the five discussed solution methods. Under all scenarios, the tests were run for various constraints on the number of clinics where *P* is increased between 1 and the maximum number of nodes available. The results of this analysis can be seen in Table 1.

**Table 1:** Scenario 1 results—Restricted to existing facilities.

Scenario	Algorithm	Number of runs	Optimal found %	Average CPU time (seconds)	Optimal coverage (Total %)	Number of selected facilities
1a	Greedy	-	-	0.087	91%	25
1a	Gurobi	-	-	10.835	91%	25
1a	GA	100	100%	0.982	91%	25
1a	TS	100	100%	1.029	91%	25
1a	SA	100	100%	0.061	91%	25
1b	Greedy	-	-	0.028	99%	18
1b	Gurobi	-	-	4.420	99%	14
1b	GA	100	100%	0.987	99%	14
1b	TS	100	100%	0.181	99%	14
1b	SA	100	100%	0.104	99%	14

**Fig 3:**
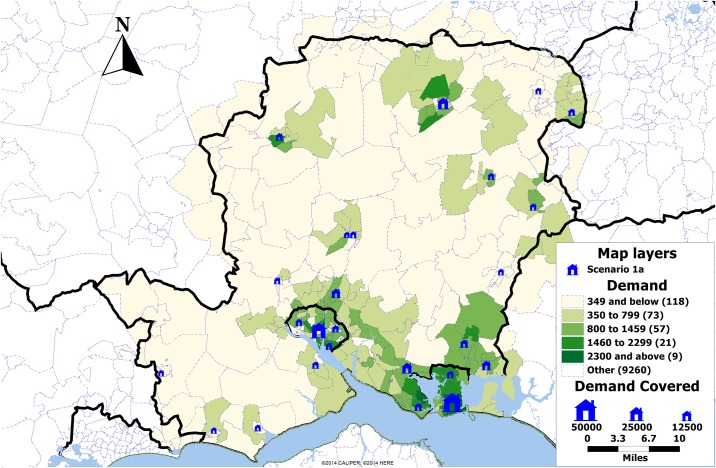
Map showing the demand by postcode sector in Hampshire, UK, and the locations selected in scenario 1a (blue). The size of the clinics is proportional to the demand expected over the period of one year.

**Fig 4:**
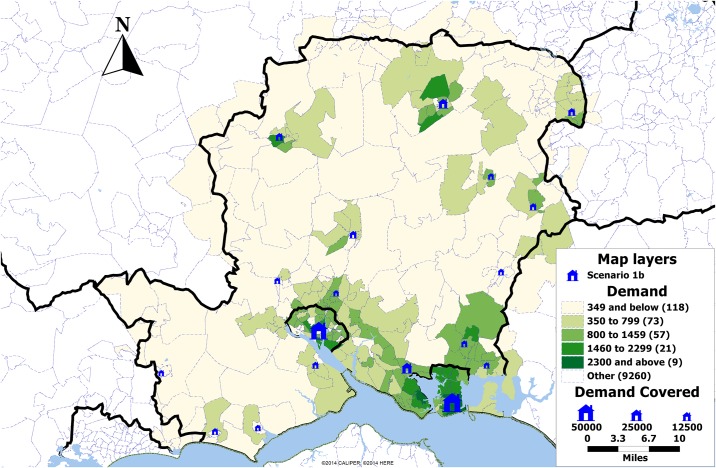
Map showing the demand by postcode sector in Hampshire, UK, and the locations selected in scenario 1b (blue). The size of the clinics is proportional to the demand expected over the period of one year.

Table 1 shows the number of selected location for the maximum coverage under each scenario. Under scenario 1a all five solution methods result in the same set of locations covering 91% of the population (Fig 3). Additionally, under scenario 1b the greedy solution results in 4 extra clinics as compared to the search method solutions where 14 clinics are selected while all solutions provide a population coverage of 99% (Fig 4).

In the second scenario, we are allowing a facility to be located anywhere within the county boundaries. That is any of the current clinic locations in addition to all the postcode sectors within the area. This results in a set of 305 candidate locations. The result of this scenario can be seen in Table 2.

**Table 2:** Scenario 2 results—Candidate location permissible any postcode sector centroid.

Scenario	Algorithm	Number of runs	Optimal found %	Average CPU time (seconds)	Optimal coverage (Total %)	Number of selected facilities
2a	Greedy	-	-	0.091	188054 (100%)	61
2a	Gurobi	-	-	52.924	188054 (100%)	59
2a	GA	100	100%	404.470	188054 (100%)	70
2a	TS	100	100%	87.974	188054 (100%)	75
2a	SA	100	100%	6.487	188054 (100%)	59
2b	Greedy	-	-	0.068	188054 (100%)	19
2b	Gurobi	-	-	12.731	188054 (100%)	15
2b	GA	100	100%	279.926	188054 (100%)	26
2b	TS	100	100%	222.817	188054 (100%)	25
2b	SA	100	100%	2.861	188054 (100%)	18

**Fig 5:**
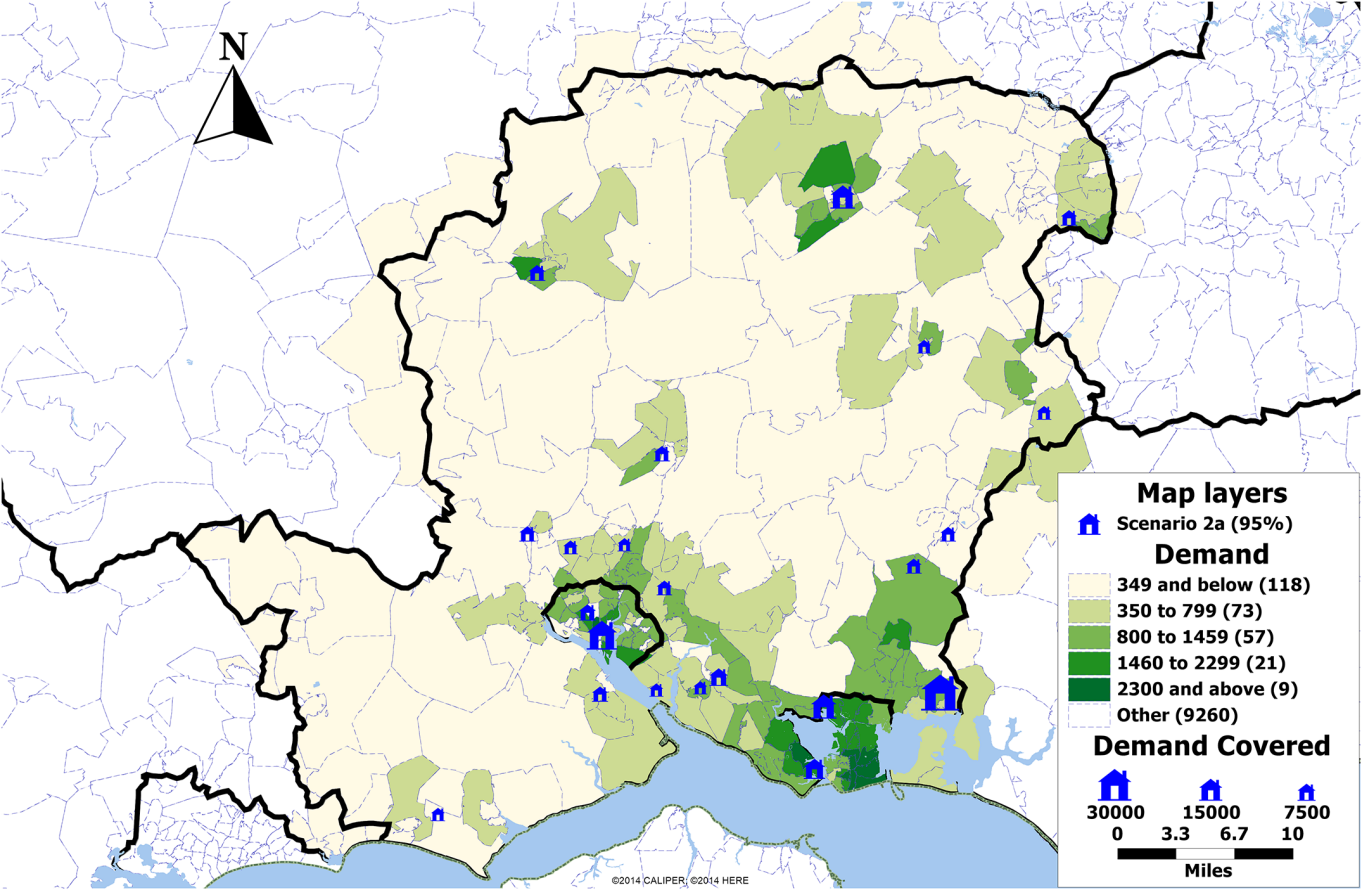
Map showing the demand by postcode sector in Hampshire, UK, and the locations selected providing 95% coverage in scenario 2a (blue). The size of the clinics is proportional to the demand expected over the period of one year.

**Fig 6:**
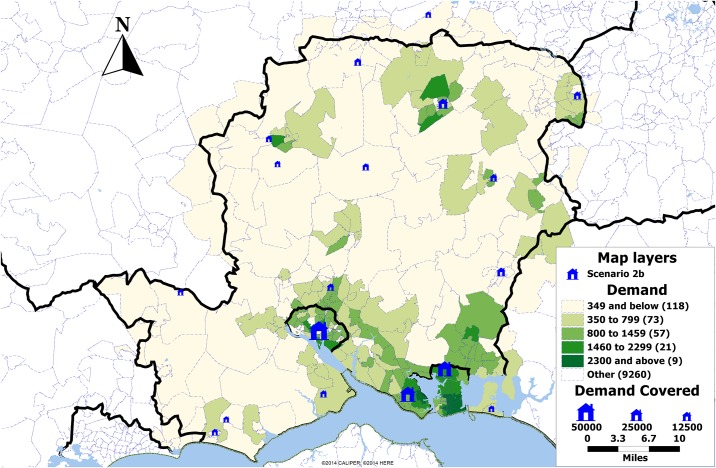
Map showing the demand by postcode sector in Hampshire, UK, and the locations selected in scenario 2b (blue). The size of the clinics is proportional to the demand expected over the period of one year.

Under scenario 2a, in all four cases the initial 60% of population is covered by the same locations while there is some minor variation in selection of further locations. This could be explained by the fact that many postcode sectors are close to each other so one could be chosen instead of another, resulting in slightly different set of selected locations. Under this scenario, exact solution set provides the solution with 60 locations chosen (Fig 5). The same is true for scenario 2b, where a solution set of only 19 clinics is selected (Fig 6).

It should be noted as the driving times are much lower than public transport times it comes as no surprise that a lower number of locations are selected. Additionally, it is clear that when there is no restriction on the location of the facilities the locations are chosen so 100% of the population coverage is achieved.

**Fig 7:**
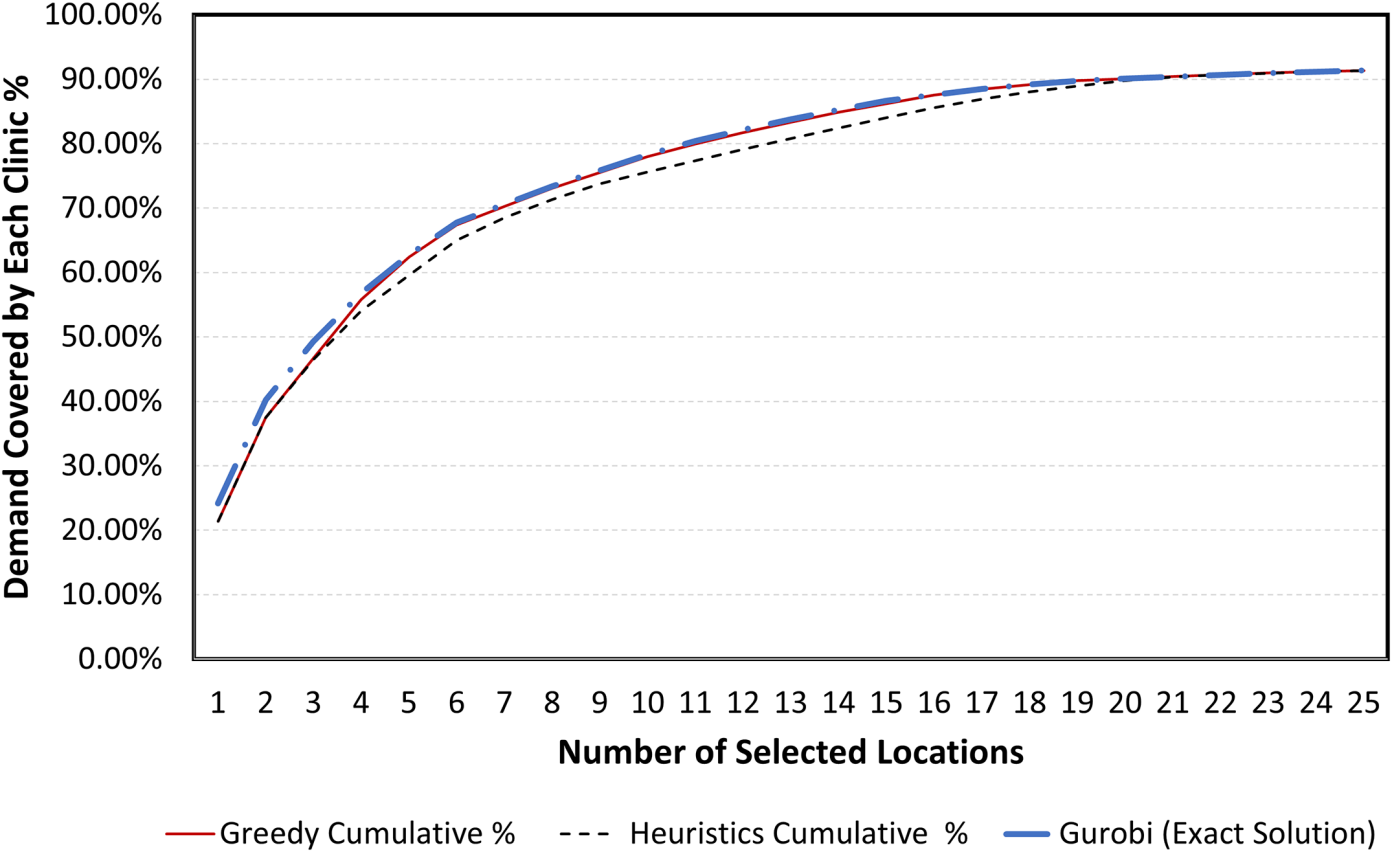
Demand coverage when candidate locations restricted to existing facilities (scenario 1a).

Furthermore, as the number of chosen clinics increases, the demand covered diminishes after a certain point. This effect can be seen in Fig 7. The trade off curve, solid and dashed lines in Fig 7 shows the maximum possible coverage within the desired travel time for various number of facilities. The same could be seen in S1, S2 and S3 Figs for other scenarios.

## Discussion

Based on the solution found by the greedy algorithm under scenario 1a, 20 facilities may be positioned to achieve coverage of 90% of the population, but 25 facilities may be required for full coverage (see Table 3). The decision maker may view the required additional funds as money which could be spent in other beneficial ways than achieving full coverage within the desirable travel time. It should be noted, that under this scenario only 91% of total demand is covered. This is as a result of only selecting from the set of current clinics locations.

**Table 3:** Result comparison by percentage of covered demand.

Scenario	Number of Clinics to Cover 90%	Number of Clinics to Cover 100%
1a (All Algorithms)	20	25
1b (Ga, Tabu & SA)	8	14
2a (Gurobi)	17	60
2b (Gurobi)	7	18

**Fig 8:**
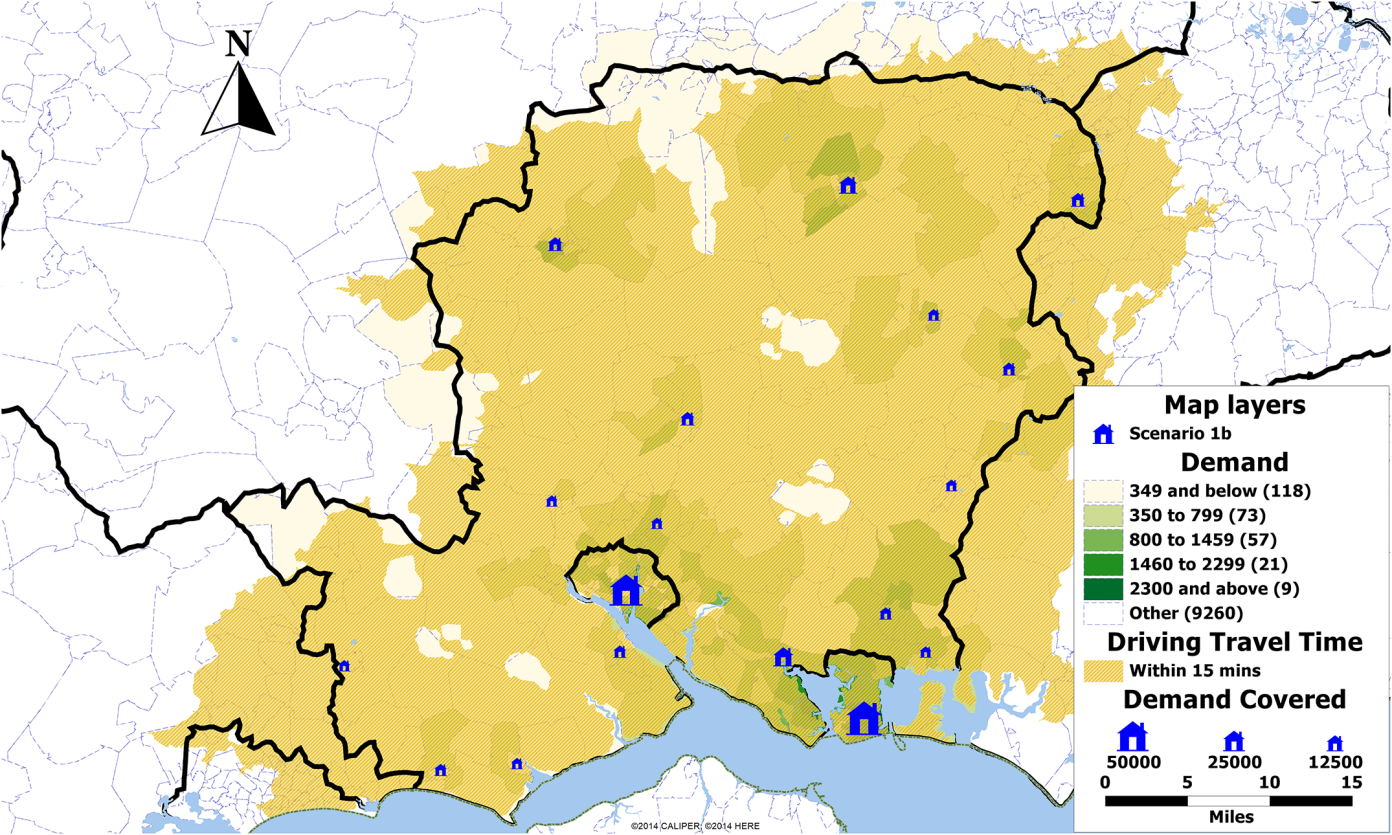
Map showing the non-aggregated 15 minutes driving time coverage by scenario 1b. The size of the clinics is proportional to the demand expected over the period of one year.

**Fig 9:**
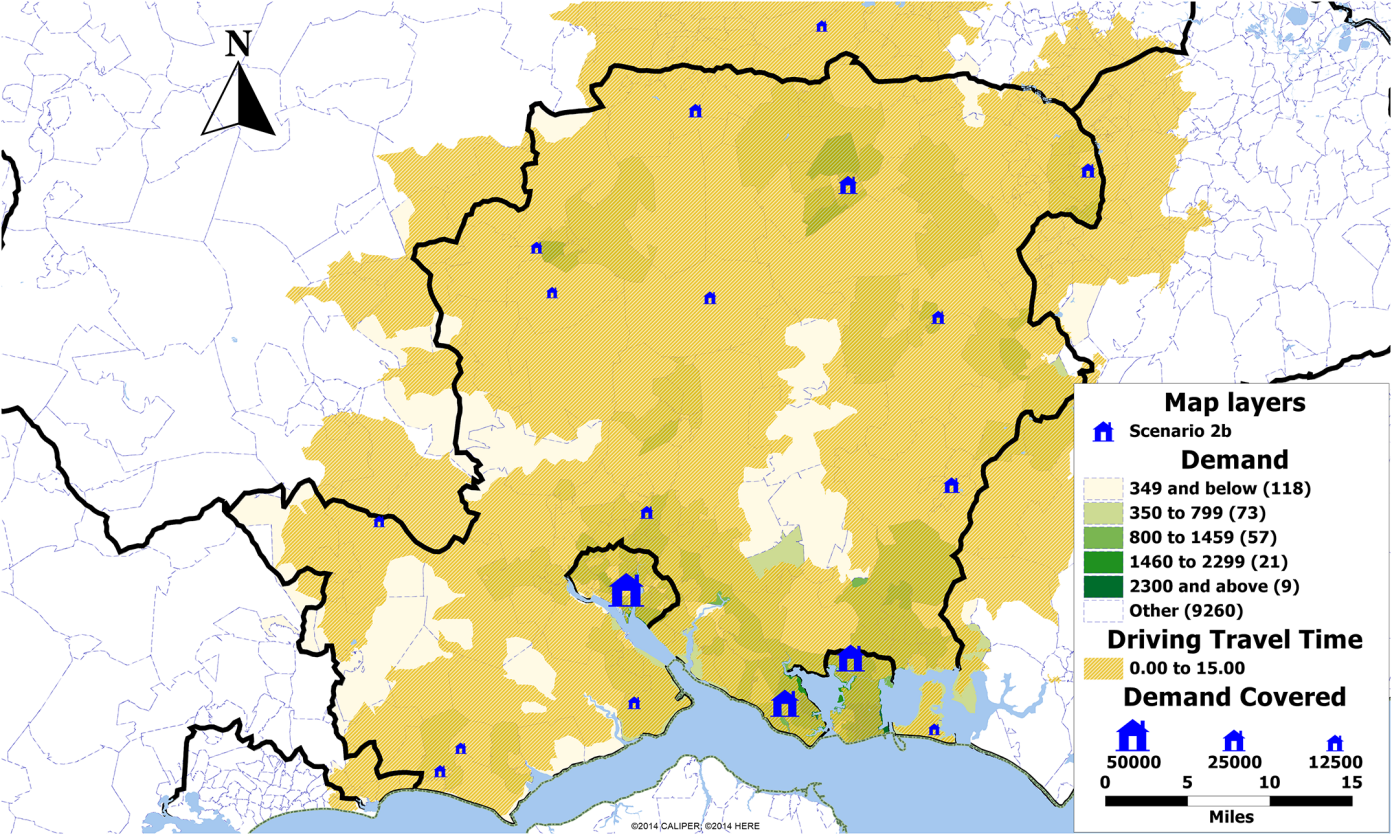
Map showing the non-aggregated 15 minutes driving time coverage by scenario 2b. The size of the clinics is proportional to the demand expected over the period of one year.

It is well documented that the level of aggregation has a direct effect on the computation times and the usefulness of the analyses [30, 31]. While in this paper we were limited by the availability of full postcode due to governance, we have taken measures to check the feasibility of a subset of solutions, specifically scenarios 1b and 2b. Figs 8 and 9 show the coverage within 15 minutes of driving time for each scenario respectively. It can be seen from the maps, that only very small pockets are not covered by any clinics within 15 minutes when the travel nodes are not aggregated. It should be noted that the coverage is increased to 100% if the driving time is increased to 20 minutes in both cases.

### Strengths

When dealing with real life problems with multiple actors and agendas, results such as the above provide clear evidence that would help the decision making processes. We have used multiple solution methods to solve the facility location problem and have compared the results to a simpler algorithm that is easily understandable to health care providers and practitioners. In pursuit of this we have done our best to adhere to the standard set by the good laboratory practice for optimisation research [32].

As evident by the results in Tables 1 and 2, an exact solution can be found within seconds in all scenarios. However, for the purpose of this project an easily understandable greedy algorithm is crucial since it is more transparent and intuitive to the end user. The results show that this greedy algorithm is able to find an optimal or near optimal solution in all instances. Furthermore, there is no clear advantage in solving such location analysis problems through complex heuristic methods such as TS and GA. The SA in some cases result in slightly better solution as compared to other heuristic approaches (scenario 1b, 2a &2b), however the gain might not justify the complexity. Moreover, in scenario 2a each method results in slightly different solution while the coverage remains the same and as such the extra effort does not result in a better coverage.

Revelle et al. [33] compared the performance of metaheuristic heuristic to that of the exact solution for large scale maximal covering location problems showing that the heusristic would result in solutions no worse than 0.54% below the best known solution. Additionally, Tong el at, compared the performance of a genetic algorithm for coverage maximisation problem with other genetic algorithms as well as an exact solution showing that this algorithm is able to produce optimal or near optimal solutions. It has also been observed for set covering problems in the literature that the greedy method is surprisingly good in practice, especially when compared with other approximation algorithms [34, 35]. Few papers have carried out an empirical comparison of various solution methods for facilities location problems [36, 37]. Arostegui et al. [38] showed that statistically, SA performs similar or better than TA and GA for other types of facilities location selection problems. In general, they conclude that the performance of TS, SA and GA for the different types of facilities location problem is situational principally based on the specific problem formulation. Additionally, the search method in TS is critical to finding good solutions in practical time frames, while the stochastic search in SA is effective in avoiding cycling in the solution space. The SA finds the local minima in a more random way as compared to TS and GA. However, once a local minimum is found, SA is better able to escape the local optima and find a lower minimum [39].

The issue of favouring simple methods over more complicated ones is more crucial when the model purpose is to aid in real life decision making. Experience shows that if the stakeholders (users of the model) can easily understand the methods used, they are more likely to trust the solution and use the model more confidently in the decision making processes [40, 41].

This study provides a much needed evidence that under time pressured and resource scarce situations where the purpose of maximal coverage location analysis is to partially aid decision making, one can achieve good solutions using a simple greedy algorithm. The results from this work can be generalised for other types of health services where the aim is to improve access to facilities. However, it should be noted that further studies are needed to replicate these results in other problem settings as well as larger problem sizes.

The simple and understandable procedure and computational efficiency along with the fact that it scales up well and is capable of finding an optimal or near optimal solution makes the greedy algorithm an attractive alternative. Furthermore, given requirement for an efficient and optimal solution its best to use the exact method as is evident by the results of our cas study.

It is well documented that the level of aggregation plays an important role in discrete location selection problems. It has a direct effect on the computation times and the usefulness of the analyses [30, 31]. While in this paper we were limited by the availability of full postcode due to governance, we have taken measures to check feasibility of all solutions by calculating the coverage under each scenario by driving time.

### Limitations and further work

In any real-life facility location, there are limitations to what could be implemented. For instance, a clinic selected to be closed might not be acceptable for various practical or political reasons or a selected potential new location may not be viable due to lack of appropriate premises.

In the public transport analysis, we have assumed that all patients regardless of where they live would be travelling to a clinic by public transport. As such, an additional number of locations are selected especially in rural areas. This assumption might be valid for an urban population with access to good transport links; however, those living in rural area are more likely to have access to a car and choose to drive to the clinic of their choice. This is backed by the results of the UK 2011 Census [42] where more than 85% of households in Hampshire have access to at least 1 car with over 45% having access to more than 1 car.

The above mentioned limitation along with the variations between the results under both scenarios gives rise to the need of a tool to evaluate a user chosen set of locations. This was addressed by development of a decision making model. This model is a tool for scenario comparison, allowing exploration based on the results above. It enables stakeholders to select any number of clinic locations and investigate the coverage across the county. It calculates the projected demand of each selected clinic based on historical attendances and shortest driving time or public transport time.

The analysis carried out in this paper could be extended to take into account the locations of particular at-risk patient groups. Where information is available that certain regions are prone to higher demand for particular services, weighting can be applied to such populations. However, since such areas are usually known to the stakeholders, facilities could be manually entered into the solution through the developed decision making tool.

Thus far, we have analysed the system not taking clinic capacity into account. In reality decision makers should consider the redistribution of available treatment capacity for any given geographic configuration of estates. Further work is considering practical and transparent ways to extend our model to support capacity decisions at the same time as facility location.

## Supporting information

S1 Fig Demand coverage when candidate locations restricted to existing facilities (scenario 1b). (TIF)

S2 Fig Demand coverage when candidate location permissible any postcode sector centroid (scenario 2a). (TIF)

S3 Fig Demand coverage when candidate location permissible any postcode sector centroid (scenario 2b). (TIF)

S1 Data Anonymised data used in this project. (XLSX)

S1 Supporting information Supporting information. (DOCX)
