# Supplementary figures and images for: A facility location model for analysis of current and future demand for sexual health services
Source: PLoS One. 2017 Aug 29;12(8):e0183942. doi: 10.1371/journal.pone.0183942 (PMC5574542; doi:10.1371/journal.pone.0183942)

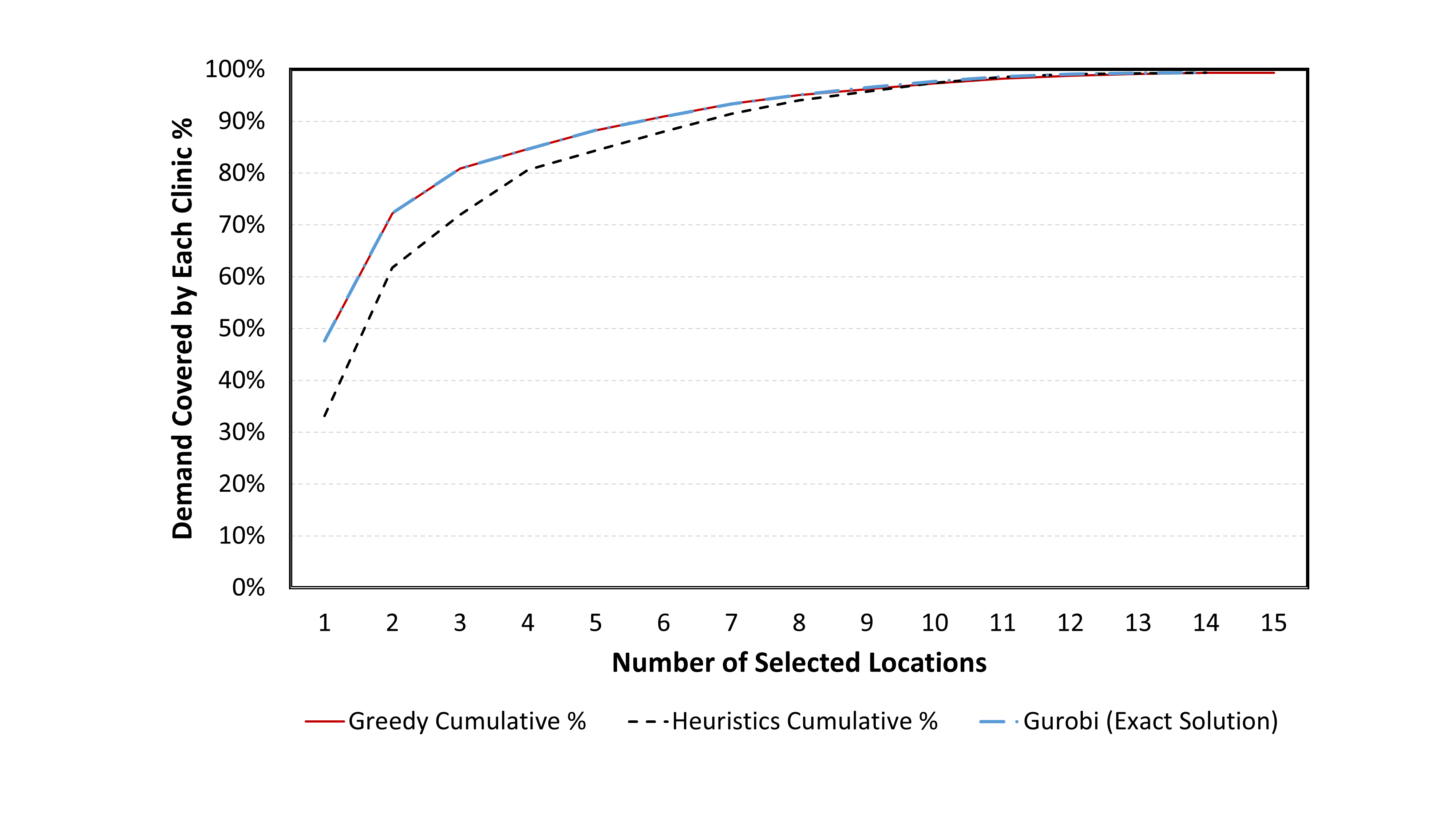

Supplement: S1 Fig — (TIF) [file pone.0183942.s001.tif]

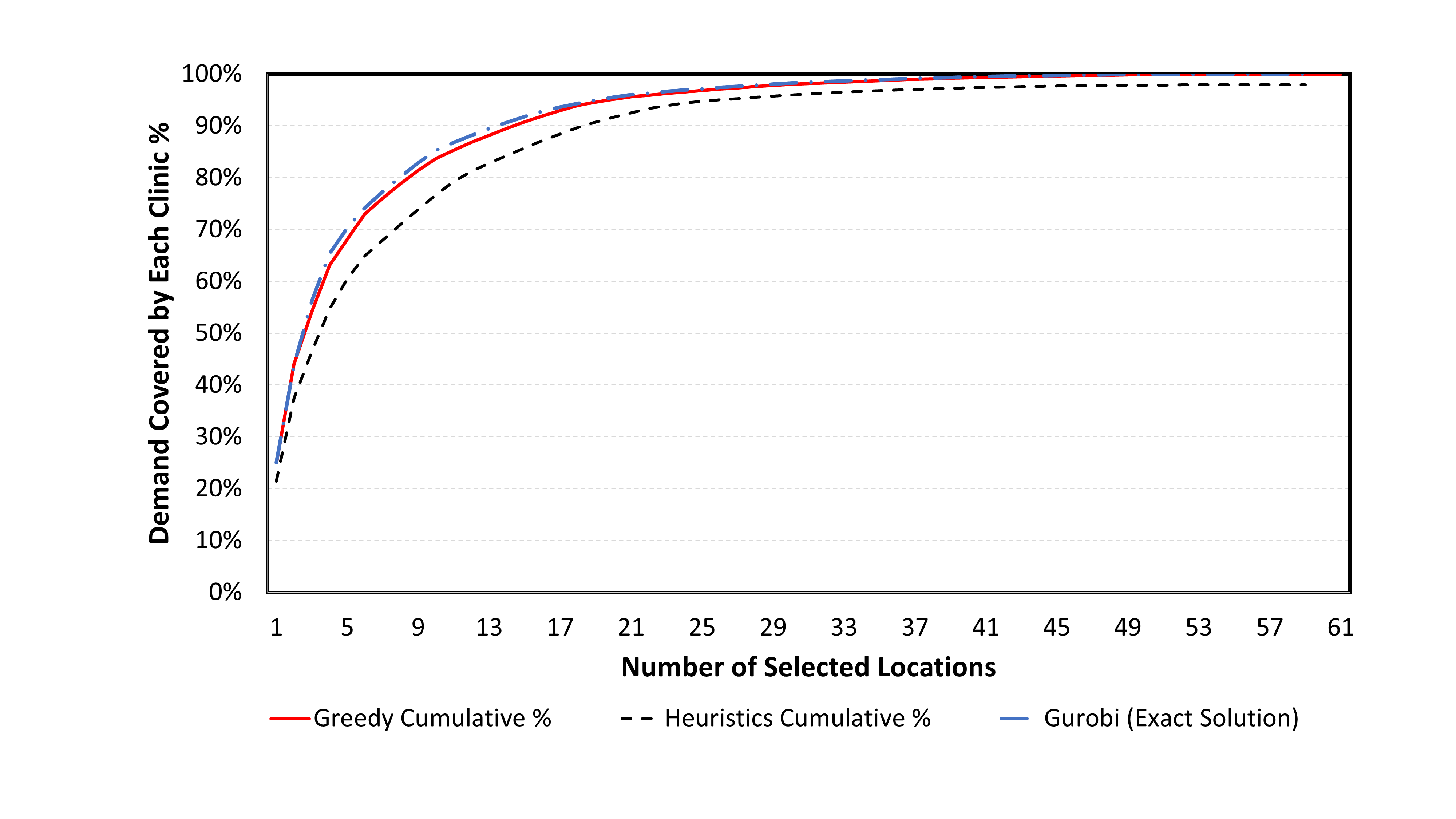

Supplement: S2 Fig — (TIF) [file pone.0183942.s002.TIF]

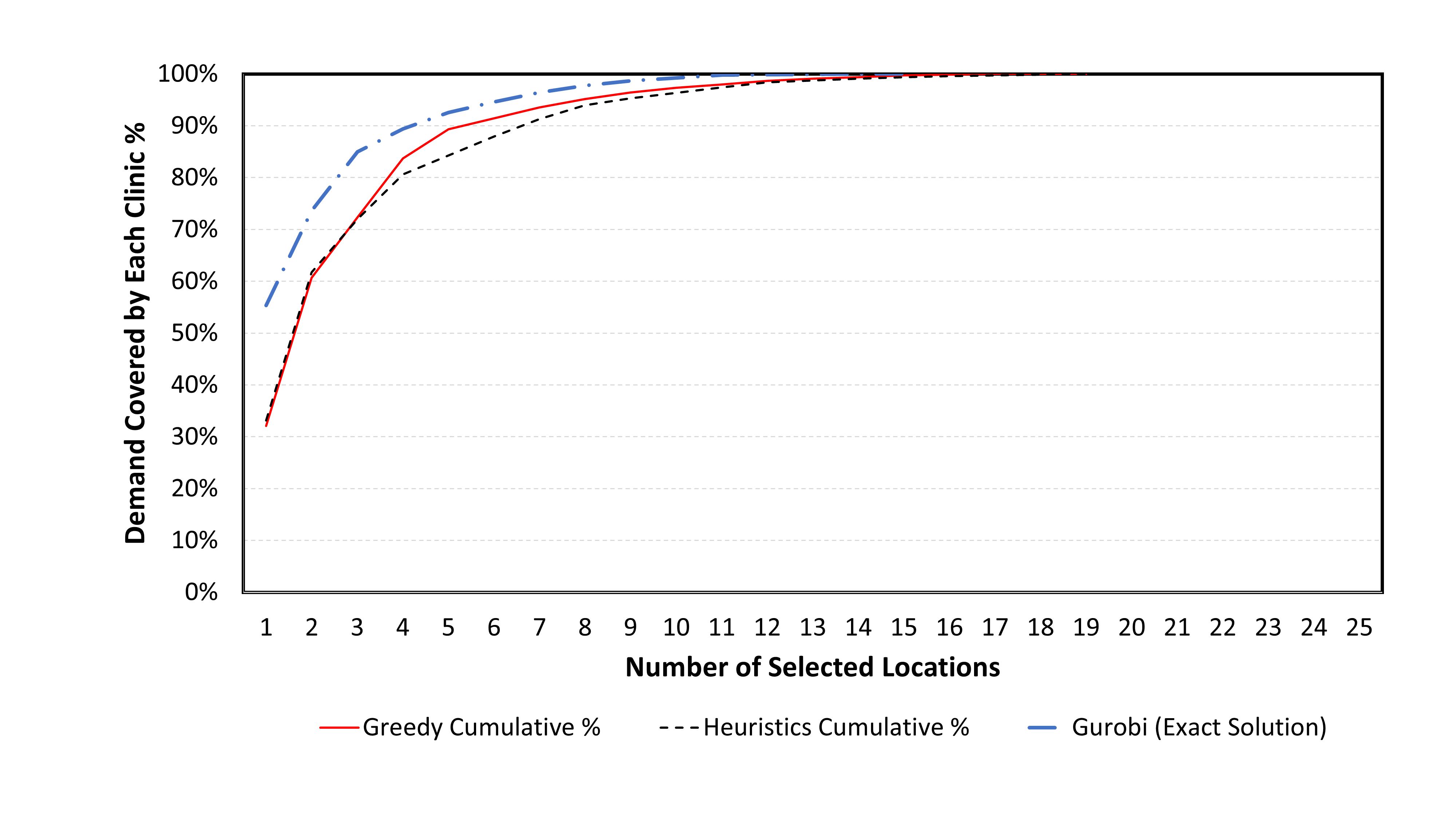

Supplement: S3 Fig — (TIF) [file pone.0183942.s003.TIF]
