# Supplementary material for: A facility location model for analysis of current and future demand for sexual health services
Source: PLoS One. 2017 Aug 29;12(8):e0183942. doi: 10.1371/journal.pone.0183942 (PMC5574542; doi:10.1371/journal.pone.0183942)
Supplement: S1 Supporting information — (DOCX) [file pone.0183942.s005.docx]

# Supporting Information

### Meta-heuristics

*Initial Solutions*

To start any of the meta-heuristics algorithms, an initial solution is required. It is preferred that this initial solution is of good quality and requires little computation time. Here, we use the Net Benefit Heuristic (NBH) algorithm which was proposed by Al-Sultan and Al-Fawzan [1] to generate a relatively good starting solution for the following genetic algorithm and the tabu search method. The NBH consists of two phases, the initial phase and refinement phase. The initial phase ensures enough facilities have been established to satisfy all demand which constitutes an upper bound on the optimal solution. The refinement phase is a procedure in which each facility that was established in the initial phase is investigated for possible closure, whereby a facility is closed if this can result in net saving. This happens when a demand point is covered by multiple facilities and closure of one would not have an effect on the coverage. Detailed description of the NBH algorithm could be found in the supplementary information.

*Generation of Neighbours*

We assume a solution is generated either at an initial stage or through algorithm iteration. Let this solution be specified by a vector$y$, where the set of all feasible solutions is denoted by$Y$, and the demand covered is denoted by $d(y)$ (the objective function). Each solution $y\in Y$ has a set of associated neighbours,$N\left( y \right)\subset Y$ called the neighbourhood of$y$. Each solution $\hat{y}\in N(y)$ can be reached directly from $y$ by an operation such as swapping, moving or replacing. In our implementation of the genetic algorithm and the tabu search, we use the scheme explained in the supplementary information for generating a neighbour $\hat{y}$ for a given location vector$y$.

*Genetic Algorithms*

Genetic algorithms (GAs) are a family of randomized-search optimization heuristics, which are based on the biological process of natural selection (see [2, 3]). GAs, are general-purpose search algorithms that correspond to natural selection’s survival of the fittest [2]. Artificial strings corresponding to chromosomes represent population members. The search starts with an initial population of strings. At each iteration individual strings are evaluated with respect to a performance criterion and are assigned a fitness value (total demand covered). Based on their fitness values, strings are selected to construct the next generation by applying the genetic operators; selection, crossover and mutation.

*Tabu Search*

Tabu Search (TS) is a local search technique that was originally developed by Glover [4]. Using an initial feasible solution TS investigates the neighbours of the existing solution in each iteration in an attempt to improve the best solution obtained so far including escaping local optima. Thus, new candidate solutions are generated by using different local search methods. In order to avoid the repetition of the same solutions, TS forbids a given number of moves by keeping these moves in a tabu list. The moves in the tabu list are not accepted unless they provide solutions better than a pre-determined aspiration level (new solution being better than the best solution so far).

*Simulated Annealing*

Simulated Annealing (SA) is one of the well-known algorithms which is inspired from physical annealing of solids (i.e. allowing metal to cool slowly in order to strengthen it). It was first introduced by Kirkpatrick [5] to solve large combinatorial optimization problems. SA attempts to escape from the local optima by probabilistically choosing non-improving solutions. It requires the construction of an initial feasible solution to the problem. SA interprets slow cooling as a slow decrease in the probability of accepting worse solutions as it explores the solution space. Accepting worse solutions is a fundamental property of metaheuristics because it allows for a more extensive search for the optimal solution.

## Genetic Algorithm

In this section, we describe in brief the GA method used in this paper. This is based on the modified GA method for the location problems considered in [3].

*Fitness Function*

The fitness function value of each possible solution to the MC problem is calculated by finding the set of open facilities belonging to the solution being evaluated and adding the demands that are covered by these facilities. Care must be taken in order to avoid demands being counted several times since one demand point might be covered by several facilities at the same time.

*Parents’ selection-procedure*

In this paper we chose the Binary Tournament Selection Method (see Beasley and Chu [6]) because (i) it can be implemented very efficiently, and (ii) in Beasley and Chu [6] it has been shown that this method gives solutions whose quality compare favourably to the ones produced by other methods [3].

*Crossover operator*

We use one-point crossover operator as it is simple to implement. To tackle the possibility of getting an offspring identical to one of its parents we have incorporated a mutation operator within the crossover operator.

Mutation helps to prevent solutions from being trapped in local optima. In this work, the mutation operator works by selecting randomly one of the open facilities and moving this to another site. The new site is also picked randomly from the set of empty possible places to locate facilities.

*Population size and replacement population method*

Here we use the commonly known method is the generational replacement, which generates a new population of children and replaces the whole parent population (see Beasley et al. [6]).

One of the most obvious questions relating to GA performance is how it is influenced by population size. It is clear that small populations run the risk of seriously under-covering the solution space, while large populations incur severe computational penalties. Here we have used 2n where n is the number of possible facility sites as suggested in [3].

Thus, the GA that we implemented can be described algorithmically as follows:

Set t: =0;

1. Generate initial population $P(t)$ (See NBH and neighbourhood generation algorithm)
2. Evaluate each of the $P(t)$members according to the total demand covered
3. While (No. generations $\leq$ max value or improvement in objective function $\leq{10}^{-5}$

Do

1. Set $t:= t+1$
2. Select solutions P1 and P2 from the $P(t)$ using binary tournament selection
3. Apply genetic operators to P1 and P2
   - Apply the one point crossover operator
   - Compare child C1 with its parents. If it is not identical to either then got to next step. Otherwise apply mutation to the parent with the lesser fitness to form a child C1.
4. Repeat steps 6 & 7 until a new set of children is created which is of the same size as the parent population.
5. Evaluate this new child set according to the kind of problem being solved
6. Create $P(t)$ by replacing it with the new child set.

## Initial Generation of Neighbourhood

An initial solution is required before one that start the genetic algorithm and tabu search algorithm. To ensure that this initial solution is of high quality we use the net benefit heuristic (NBH) to generate an initial feasible solution (for more details on NBH see [1]).

*The NBH Algorithm*:

Initial Phase:

1. For each demand $j, j=1,2,\ldots N,$find the facility that can supply this demand for the cheapest cost and denote it by $i*$, form set $V(i*,j)$
2. Form the set $I$ from $V$ by extracting the first index. This is the list of suggested facilities
3. Evaluate the current solution which constitutes an UB on the optimal solution

Refinement:

1. Set $k=1$, let $m*$ be number of suggested facilities
2. Consider the $kth$ facility established in the initial phase. Let $i$be the kth element in$I$.
3. Let the set $J$ be the indices of all those demands currently covered by facility$i$.
4. For each demand currently covered by *i*, find which established facility other than *i* can satisfy this demand at min cost and compute the extra cost due to this reallocation
5. Compute the extra cost which is equal to the cost of reallocation of demand, less the saving due to the closure of facility *i*.
6. If the extra cost is negative, then it is better to close the facility (go to next step). Otherwise consider the next facility.
7. $I=I-\{i\}$. UB=UB- extra cost. If m=1 stop; otherwise go to next step.
8. If$k=m*$, stop; otherwise, set$k=k+1$ and go to step 2.

*Neighbourhood Generation Algorithm:*

Let $y_{i}$ be the$ith$ component of the given vector y, where $1\leq i\leq n$ and n is the number of facilities. To generate a neighbour $\hat{y},$ perform the following steps:

1. Let $\alpha$be a given constant, where$0<\alpha<1$. $\alpha$is considered a parameter of this scheme (Probability threshold)
2. Set$k=1$.
3. Generate a random number$\beta\in U(0,1)$.
4. If$\beta>\alpha$, go to step 5; otherwise, go to step 6.
5. If$y_{k}=1$, set$\hat{y}_{k}=0$, and if$y_{k}=0$, set$\hat{y}_{k}=1$; stop.
6. If$k=n$, stop. Otherwise set $k=k+1$ and go to step 3.

## Tabu Search Algorithm

Choose nbhsize (neighbourhood size), tabu list size, Itermax (max non improving iter). Let$k=1$:

1. Generate nbhsize random solutions from$y_{current}$. Each solution is evaluated and the solution with minimum total cost among the generated solutions is selected as $y_{min}$ (see NBH and neighbourhood generating algorithm).
2. Check whether or not the solution $y_{min}$ found is step 1 is in the tabu list. If $y_{min}$is not in tabu list or it is but the min < BV, then go to step 3. Otherwise let the best solution so far be $y_{min}$ and repeat this step.
3. Update current solution; Store $y_{min}$ in tabu list if min $\geq$ BV, then go to step 4; otherwise,$BV=min$, $k=0$, and go to step 4.
4. Check stopping criteria: if $k=IterMax$ go to step 5, otherwise $k=k+1$ and perform another iteration.

## Simulated Annealing

The SA method used in this paper is a modified version of the algorithm presented in [7] for large set covering problems.

Define:

MT: Max Iteration Number;

T: Initial Temperature;

TL: Temperature length; the No. Iterations at a particular value of T;

CF: Cooling factor; the % by which T is reduced after TL iterations;

Construct a feasible solution S and Calculate Z(S) (see *Simulated Annealing Initial Solution)*

Do while Iteration < MT

Search for a neighbour S’ of S (see *Simulated Annealing Search Module)*

1. Let $Z(S’)$ be the cost $S’$ and t $\delta=Z(S’) - Z(S)$;
2. If $\delta\leq0$, let $S=S’$ and $S^{*}=S’$; otherwise let $S=S’$ with probability $e^{-\frac{\delta}{T}} ;$
3. Let $T=T \times CF$;
4. If maximum iteration is reached return the optimum solution $S^{*}$ and$Z(S^{*})$; otherwise go to next iteration.

*Simulated Annealing Initial Solution*

Define:

- R: the set of all locations not in the solution.
- S: the set of all locations in the solution.
- $w_{i}$: the number of locations covering$i, i\in I$.
- U: the set of uncovered demands (i.e. rows with $w_{i} =0).$

Initialise:

Set$R=J, S=\{\emptyset\}, U=I, w_{i}=0$, for all $i\in I$

1. Randomly select a row $i, i\in U.$
2. Select the first column (location) $j, j\in R$, for which $a_{ij}=1.$ Move column *j* from *R* to *S*.
3. Set $w_{i} = w_{i} +a_{ij}$ for all$i\in I$. Define *U* as the set of rows for which$w_{i} =0$. If *U* is empty, go to step 4, else return to step 1.
4. Examine each column, *k*,$k\in S$ in reverse order. If $w_{i} - a_{ik} \geq1$ for all$i\in I$, then move *k* from *S* to *R* and set $w_{i}=w_{i} - a_{ik}$ for all$i\in I$.

**Let:**

*Z(S)* = the total solution cost;

*N(S)* = the number of selected locations (columns) in set *S*;

$Q\left( S \right)= max\left( c_{j} \right|j\in S)$; the maximum location cost in set *S*

*Simulated Annealing Search Module*

Set $d=0, D=[\rho_{1} N(S)]$ and $E=[\rho_{2}Q(S)]$

1. Randomly select a column $k, k\in S$
2. Move *k* from *S* to *R*. set$w_{i}=w_{i} - a_{ik}$, for all$i\in I$. Set$d=d+1$. If$d=D$, go to step 3, otherwise, return to step 1.
3. Define *U* as the set of rows for which$w_{i}=0$. If U is empty, then go to step 6, otherwise go to step 4.
4. Define $R_{E}$ as the set of columns for which$c_{j}\leq E | j\in R$. Compute the following:
   - $\alpha_{ij}=1$ if $w_{i}=0 and \alpha_{ij}=1$ for all $i\in I, J\in R_{E}$;0 otherwise
   - $\alpha_{j}=\sum_{\left\{ i\in I \right\}} \alpha_{ij}$ for all $j\in R_{E}$;
   - $\beta_{j}=c_{j}\alpha_{j}$
   - $\beta_{min} = min_{j}\left( \beta_{j} \right|j\in R_{E})$
   - *K=* set of columns for which$\beta_{j} =\beta_{min} | j\in R_{E}$.
5. Randomly select a column k/in K and move that column from *R* to *S*. Let$w_{i}=w_{i} + a_{ik}$. Return to step 3.
6. Examine each column, *k*$, k\in S$, in reverse order. If $w_{i} -a_{ik}\geq1$ for all$i\in I$, then mover *k* from *S* to *R* and set$w_{i}=w_{i} - a_{ik}$, for all$i\in I$.

Table 4. Initial parameters used in the analysis based on the literature.

| **Algorithm** | **Max non-improving iteration** |
| --- | --- |
| Genetic Algorithm (GA) [3] | Neighbourhood size: 2n  Max non-improving iteration:5  $\alpha$=0.9 |
| Tabu Search (TS) [3] | Neighbourhood size:2n  Max non-improving iteration:5  $\alpha$=0.9 |
| Simulated Annealing (SA) [7] | Initial temperature: 1.3  No. iteration at particular temperature:100  Cooling factor:0.9  Max non-improving iteration:10  $\rho_{1}$=0.4  $\rho_{2}$=2 |

# References

1. Al-Sultan, K.S., A Al-Fawzan, M., *A tabu search approach to the uncapacitated facility location problem.* Annals of Operations Research, 1999. **86**: p. 91-103.

2. Goldberg, D.E., *Genetic algorithms in search, optimization and machine learning*. 1989, Reading, MA: Addison-Wesley.

3. Jaramillo, J.H., Bhadury, J., Batta, R., *On the use of genetic algorithms to solve location problems.* Computer & Operations Research, 2002. **29**: p. 761-779.

4. Glover, F., *Heuristic for integer programming using surrogate constraints.* Decision Sciences, 1977. **8**: p. 156-166.

5. Kirkpatrick, S., Gelatt, S. D., M. P. Vecchi, M. P., *Optimisation by Simulated Annealing.* Science, 1983. **220**(4598).

6. Beasley, J.E., Chu, P.C., *A genetic algorithm for the set covering problem.* European Journal of Operational Research, 1996. **94**: p. 392-404.

7. Jacobs, L.W., Brusco, M. J., *Note: A local search heuristic for large set-covering problems,.* Naval research logistics, 1995. **42**: p. 1129-1140.
